# Revision of *Gyrodactylus salaris* phylogeny inspired by new evidence for Eemian crossing between lineages living on grayling in Baltic and White sea basins

**DOI:** 10.7717/peerj.5167

**Published:** 2018-07-06

**Authors:** Agata Mieszkowska, Marcin Górniak, Agata Jurczak-Kurek, Marek S. Ziętara

**Affiliations:** Department of Molecular Evolution, Faculty of Biology, University of Gdańsk, Gdańsk, Poland

**Keywords:** *Gyrodactylus salaris* phylogeny, Molecular clock, Gyrodactylosis

## Abstract

In this research, grayling-specific *Gyrodactylus salaris* Malmberg, 1957 isolates from Baltic Sea basin were collected in Sweden for the first time. Samples were obtained in three drainage systems: Kalixälven (River Kaitum), Ljungan (River Sölvbacka strömmar), and Umeälven (River Juktån). Three molecular markers were analysed: nuclear ITS rDNA (Internal Transcribed Spacer) and ADNAM1 (Anonymous DNA Marker 1), and mitochondrial *cox*1 gene. As a result, four new mitochondrial haplotypes were identified (III-C1tt, III-C1tt_ht_, IX-A1tt and X-A1tt). The ADNAM1 analyses resulted in revealing two new alleles (WS4 and BS9) and two new genotypes (T6 and T7). T7 seems to be an indicator of ancient crossing between Baltic and White Sea lineages of the parasite which happened during a first 3000-year period of Eemian interglacial about 130,000 years ago in the connection between Baltic and White Sea. Molecular clock estimates were adjusted, revealing the mean substitution rate and the divergence rate among branches of 3.6% (95% HPD: 2.2%–5.2%) and 7.2% per million years, respectively. As a result, *cox*1 phylogeny rooted with the introgressed haplotypes has been revised and altered in accordance to new data, revealing fourteen equidistant lineages five of which have been excluded from the study. Based on the new phylogenetic approach, including the molecular clock, this work suggests an overall revision of *G*. *salaris* phylogeny and attempts at precisely drawing the division of lineages within this polytypic species as well as proposes unification in nomenclature for its strains.

## Introduction

Viviparous gyrodactylids are fish ectoparasites widely distributed all over the world. The most infamous of them, *Gyrodactylus salaris* Malmberg, 1957, caused thousands of tons of Atlantic salmon *Salmo salar* (L.) to die in the 1970s in Norway (Johnsen & Jensen, 1992). It resulted from introducing the parasite from the Baltic Sea basin into Norwegian rivers (Meinilä et al., 2004). The estimated economical losses caused by this epidemic equalled 480 million Euro (Hansen et al., 2004). Another epidemic caused by Baltic strains of the parasite was observed in the River Keret’, Russian Karelia in late 1990s (Kudersky, Ieshko & Schulman, 2003), and it is known to have been triggered around 1992, when *G. salaris* was first identified in the area (Ieshko & Schulman, 1994; Schulman, Ieshko & Shchurov, 1998). The infestation spread so fast that in only four years it led to a 200-fold reduction in the Atlantic salmon parr density in the Keret’ river (Schulman, Ieshko & Shchurov, 1998) with prevalence reaching the level of 100 in some of the Keret’s tributaries within two years of the infection (Kudersky, Ieshko & Schulman, 2003). The latest severe epidemic was reported in an Estonian fish farm. The infection spread within one week with prevalence reaching 100% and intensity rate much higher than normally observed in salmonid fish farm populations. The infection also proved to have been drastically more aggressive on triploid fish (Ozerov et al., 2010).

*G. salaris* is known to be polytypic and consist of several lineages identified by mitochondrial *cox*1 sequences (Hansen, Bachmann & Bakke, 2003; Meinilä et al., 2004; Hansen, Bakke & Bachmann, 2007; Kuusela, Ziętara & Lumme, 2007). It also includes the lineage specific to grayling *Thymallus thymallus* (L.) from river Hnilec formally described by Žitňan (1960) as *Gyrodactylus thymalli* (see Hansen et al., 2006; Ziętara et al., 2010) which has recently been synonymized with *G. salaris* (Fromm et al., 2014). These lineages form several equidistant clades that correspond to the host and its location; however, the phylogeny of *G. salaris* is not yet complete. Hansen, Bakke & Bachmann (2007) have demonstrated that addition of six new haplotypes from grayling-infecting strains may extend the phylogenetic tree by five clades proving that many local, potentially infectious *G. salaris* strains are still unknown. This is especially true for *G. salaris* infecting grayling, which is considerably harder to catch.

In this work, for the first time grayling-specific *G. salaris* isolates from Baltic Sea basin were collected in Sweden. Four new haplotypes were identified with the use of one mitochondrial (*cox*1 gene) and two nuclear (Internal Transcribed Spacer of ribosomal DNA, ITS rDNA and Anonymous DNA Marker 1, ADNAM1) markers which guaranteed accurate identification of the species strains. All strains found were unique for Sweden. Consequently, this work attempts at revising *G. salaris* phylogeny and specifying lineage division of this species. What is more, it has been concluded that the need exists for unification in nomenclature for its strains, which has also been proposed herein.

## Materials and Methods

### Parasite sampling

**Figure 1:**
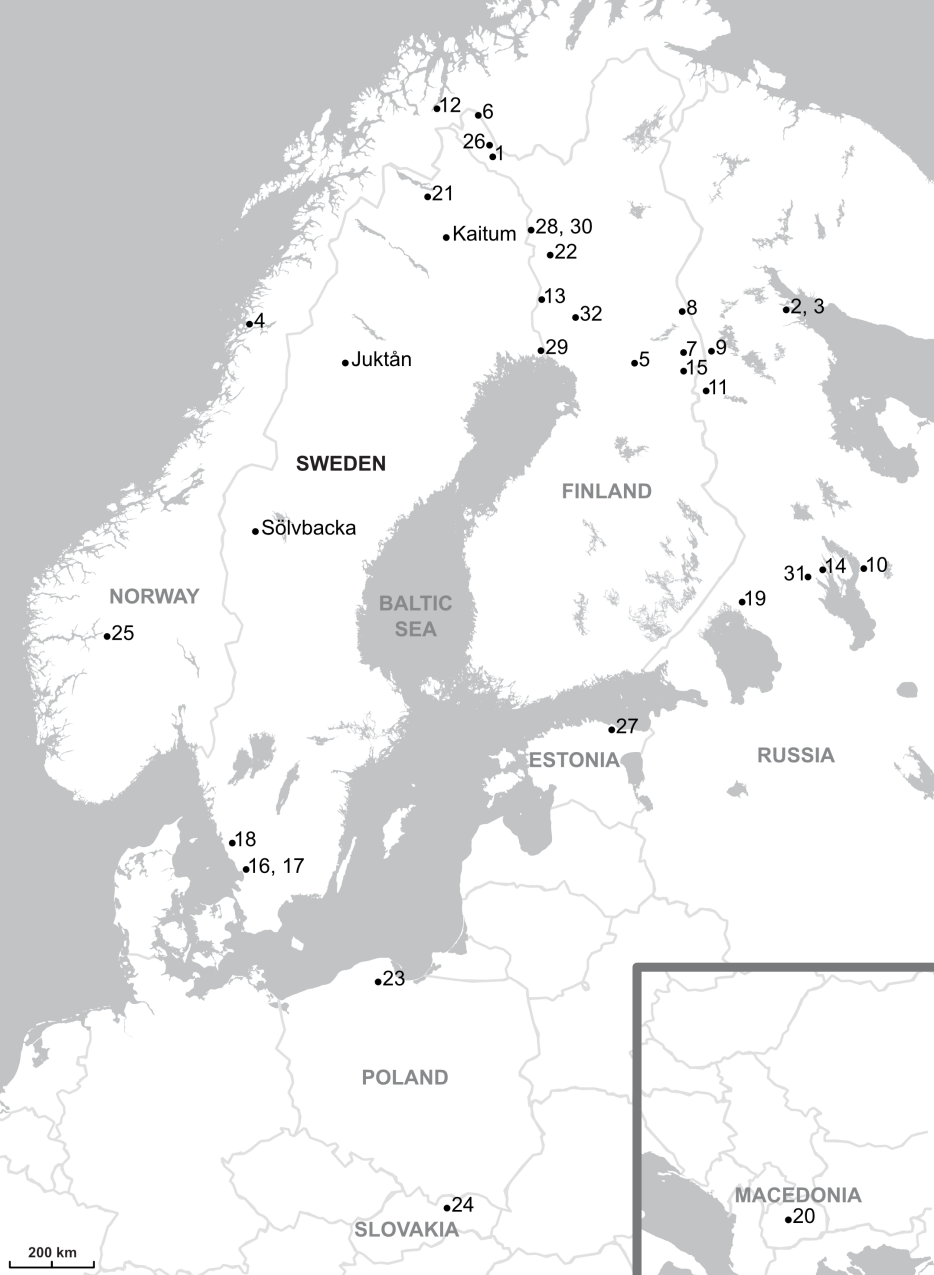
Map showing the parasites sampling localities. The sampling localities from present work are indicated by names (Kaitum, Juktån, Sölvbacka). The numbers refer to the following published works: 1–10, Meinilä et al., 2004; 11, Ziętara, Kuusela & Lumme, 2006; 12, Huyse et al., 2007; 13–23, Kuusela, Ziętara & Lumme, 2007; 24, Plaisance et al., 2007; 25, Ziętara, Johnsen & Lumme, 2008; 26, Kuusela et al., 2009; 27, Ozerov et al., 2010; 28–32, Lumme et al., 2016. Map credit: OpenStreetMap contributors.

Wild graylings (total body length 30–49 cm) were caught by means of fly fishing in three Baltic Sea drainages in Sweden: River Kaitum (Kalixälven drainage basin) on 30 July 2011–five specimens, River Sölvbacka strömmar (Ljungan drainage basin) on 5–10 July 2013–13 specimens, and River Juktån (Umeälven drainage basin on 19 July 2013–12 specimens (Fig. 1, Table 1). The grayling fins were cut and preserved in 96% (v/v) ethanol. Parasites were collected in laboratory with preparation needles under a stereo-microscope and preserved in 96% (v/v) ethanol. Opisthaptors of all specimens were removed with a sterile scalpel blade and opisthaptoral hard parts were then used for making microscopic slides with the use of a slightly modified method by Harris et al. (1999), which allowed for better flattening of haptors, more precise morphological identification, and utilisation in additional study. The remaining body was used for DNA analysis.

**Table 1:** Sampling sites and GenBank accession numbers of the studied parasites.

Locality	No of *T. thymallusN*_infected_∕*N*_total_	No of *G. salaris*	ITS rDNA	*cox1*	ADNAM1
Kaitum 20.5711E 67.4719N	1/5	3	MG256565	III-C1tt MG273445	T3 (BS1/BS1) MG273449
		2		III-C1tt _ht_ MG273446	T6 (BS9/BS9) MG273450
Juktån 16.8637E 65.4981N	1/12	1	MG256566	IX-A1tt MG273447	T3 (BS1/BS1) MG273451
Sölvbacka strömmar 13.2693E 62.7832N	3/13	7	MG256567	X-A1tt MG273448	T7 (BS1/WS4) MG273452 BS1 MG273453 WS4 MG273454

### Molecular methods

#### DNA extraction

DNA was extracted by digesting single specimens in 10 µl of lysis solution (1 × PCR buffer, 0.45% (v/v) Tween 20, 0.45% (v/v) Igepal and 60 µg/ml proteinase K). Samples were incubated at 65 °C for 25 min and then the proteinase was inactivated at 95 °C for 10 min.

#### DNA amplification

Three DNA regions of each *G. salaris* genome were amplified: two nuclear–(ITS rDNA (Internal Transcribed Spacer) and ADNAM1 (Anonymous DNA Marker 1), and one mitochondrial–*cox*1 (cytochrome oxidase subunit 1).

The ITS rDNA region consisted of ITS1, 5.8S, ITS2. Flanking regions included: 15 bp of 18S and 9 bp of 28S rDNA. The fragment was amplified using 2-µl aliquots of lysates as PCR templates in 20 µl of reaction mixture (1 × PCR buffer, 2 mM MgCl_2_, 1 µM of each primer, 200 µM dNTPs and 0.4 U of *Taq* polymerase; Thermo Fisher Scientific, Waltham, MA, USA) (Ziętara et al., 2000). Primers used were: ITS1F (5′-GTT TCC GTA GGT GAA CCT-3′) (Ziętara et al., 2000) and ITS2LR (5′-GGT ATT CAC GCT CGA ATC-3′). The latter was newly designed for better amplification of the ITS region of the *Gyrodactylus Limnonephrotus* subgenus on the basis of ITS2R primer reported by Ziętara et al. (2000). For PCR the following profile was applied: 3 min in 95 °C, then 40 cycles (40 s in 94 °C, 30 s in 48 °C, 1 min in 72 °C) and 7 min in 72 °C. The process was finished by cooling the samples down in 4 °C.

The ADNAM1 marker was amplified in conditions identical to those applied for the ITS region, using the original primers: InsF (5′-GAT CTG CAA TTC ATC CTA AT-3′) and InsR (5′-TAC AAT TCG ACC AAG GGT AG-3′) (Ziętara, Kuusela & Lumme, 2006). These primers amplify the whole ADNAM1 fragment. For PCR, the following profile was applied: 3 min in 95 °C, then 40 cycles (40 s in 94 °C, 30 s in 48 °C, 1 min in 72 °C) and 7 min in 72 °C. The process was finished by cooling the samples down in 4 °C.

Complete *cox*1 gene was amplified using 2-µl aliquots of lysates as PCR templates in 20 µl of solution (1 × PCR buffer, 2 mM MgCl_2_, 1 µM of each primer, 200 µM dNTPs and 0.5 U *Taq* polymerase, Thermo Scientific). Primers used were: Trp1F (5′-ATATA GACGA TTTGT TTTCA-3′) and Thr1R (5′-ACAGA TTACT TGGTA TTACA-3′), both described by Kuusela, Ziętara & Lumme (2008). For PCR the following profile was applied: 3 min in 95 °C, then 37 cycles (30 s in 94 °C, 1 min in 50 °C, 75 s in 72 °C) and 7 min in 72 °C. The process was finished by cooling the samples down in 4 °C.

All PCR samples were verified in 1% agarose gel under UV light with the use of ethidium bromide.

#### Molecular cloning of ADNAM1

Molecular cloning was performed with the use of the CloneJET PCR Cloning Kit (Thermo Fisher Scientific, Waltham, MA, USA). 5 µl of ligation mixture was added to 100 µl of competent *E. coli* MC1061 (Casadaban & Cohen, 1980) and incubated on ice for 45 min. The mixture was then transferred to 42 °C for 2 min, and returned to ice for additional 2 min. Next, 1 ml of warm LB medium was added (37 °C), and the mixture was incubated in 37 °C for 1 h. The mixture was cultured on solid LA medium plates with an addition of 50 µg/ml ampicillin. Clones were analysed after 24 h of incubation.

#### Molecular species identification

For molecular identification of species, PCR-RFLP analysis of the ITS rDNA region was performed. ITS rDNA fragment from each *Gyrodactylus* specimen was amplified and the amplicons were then digested with 0.07 U/ µl HincII restriction enzyme (2 h, 37 °C) in the presence of BSA (100 µg/ml). *Gyrodactylus* species were identified on the basis of restriction patterns according to the method devised by Rokicka, Lumme & Ziętara (2007). Restriction patterns were observed under UV light after 2% agarose gel electrophoresis with ethidium bromide.

#### DNA sequencing

All of the samples were purified with the GeneJET PCR Purification Kit (Thermo Scientific). The amplicons were sequenced with the flanking and internal primers. The internal primers chosen for sequencing were: ITS1R (5′-ATT TGC GTT CGA GAG ACC G-3′) and ITS2F (5′-TGG TGG ATC ACT CGG CTC A-3′) for the ITS rDNA region (Ziętara et al., 2000); RCox4 (5′-AGA CAG GTG AAG CGA AAA CA-3′), LA (5′- TAA TCG GCG GGT TCG GTA A-3′), FCox3 (5′-GCC AAT AAC CCA ATC GTG TG-3′) for *cox*1 gene (Kuusela, Ziętara & Lumme, 2008).

The ADNAM1 region was sequenced with external primers –InsF and InsR used for amplification. All samples were sequenced commercially by Macrogen Inc. (Amsterdam, the Netherlands).

### Data analysis

All of the obtained *cox*1 sequences were initially analysed in MEGA7 (Kumar, Stecher & Tamura, 2016) and FinchTV 1.4.0. Detailed analyses were conducted in PAUP 4.0b10 (Swofford, 2002) and jModelTest 2.1.5 (Darriba et al., 2012) software. The phylogenetic hypotheses were constructed with the use of combination of Neighbor-Joining method (bootstrap 1000) and GTR+Γ+I. Furthermore, Maximum Composite Likelihood and Kimura’s 2-parameter distances were compared. GenBank sequences were also utilized (Table 2). Only the complete *cox*1 sequences were finally used in the phylogenetic analyses. To analyse the ADNAM1 marker, a parsimonious network utilized by Kuusela, Ziętara & Lumme (2007) was manually reconstructed.

**Table 2:** Variable sites of *G. salaris* within the ADNAM1 marker (Anonymous DNA Marker 1).

	Variable site											
Haplotype	Del 57–79	Del 125–146	209	214	244	245	250	273	302	316	317	346
BS1 DQ468136^a^	−	−	T	C	G	A	T	C	G	T	A	T
BS2 DQ667946^a^	−	−	T	A	G	A	T	C	G	T	A	T
BS3 DQ667944^a^	−	−	A	C	G	A	T	C	G	T	A	T
BS4 DQ468130^b^	−	−	T	C	G	A	T	C	A	T	A	T
BS5 DQ667958^a^	−	−	T	C	G	A	T	T	G	T	A	T
BS6 DQ468132^a^	−	−	T	C	G	A	T	T	G	T	A	G
BS7 DQ667955^a^	−	−	T	C	G	G	T	T	G	T	A	T
BS8 DQ436477^b^	+	−	T	C	G	A	T	C	G	T	T	T
BS9 MG273450^c^	−	+	A	C	G	A	T	C	G	T	A	T
WS1 DQ468135^a^	−	−	T	A	G	G	T	C	A	A	A	T
WS2 DQ667949^a^	−	−	T	A	G	G	−	C	A	A	A	T
WS3 DQ667960^a^	−	−	T	A	G	G	T	C	A	T	A	T
WS4 MG273454^c^	−	−	T	A	C	G	T	C	A	A	A	T

**Notes.**

a Kuusela, Ziętara & Lumme, 2007

b Ziętara, Kuusela & Lumme, 2006

c Present study.

The *cox*1 matrix was used to estimate the divergence time within the *G. salaris* strains. The Bayesian uncorrelated relaxed molecular clock approach implemented in BEAST 1.8.1 (Drummond et al., 2012) was used. The divergence time estimates were based on the assumption that the mitochondrial haplotypes of the salmon-specific lineage I (SalBa) got spatially isolated about 132,000 years ago, after two *G. salaris* lineages living on grayling *T. thymallus* had crossed and switched host from grayling to salmon. The subsequent divergence created a separate, monophyletic salmon-specific clade I (Meinilä et al., 2004; Kuusela, Ziętara & Lumme, 2007), and this comprises the chosen calibration point.

The Yule process was chosen for speciation and the Akaike Information Criterion implemented in ModelTest v.3.7. (Posada & Crandall, 1998) was used to identify the best-fit evolutionary model for *cox*1—GTR+Γ+I. Calculations were performed in BEAST–each run set for 10 million generations and sampling frequency of 100. Log files were analysed with the use of Tracer v.1.6 (Rambaut et al., 2014) to assess the convergence and confirm that the combined effective sample sizes for all parameters were larger than 200. All resulting trees were then combined with LogCombiner v1.7.3 (Drummond & Rambaut, 2007), with a burn-in of 25%. A maximum credibility tree was then produced using TreeAnnotator v1.5.3 (Drummond & Rambaut, 2007) and visualised in FigTree (Rambaut, 2014).

## Results

### Species identification

**Figure 2:**
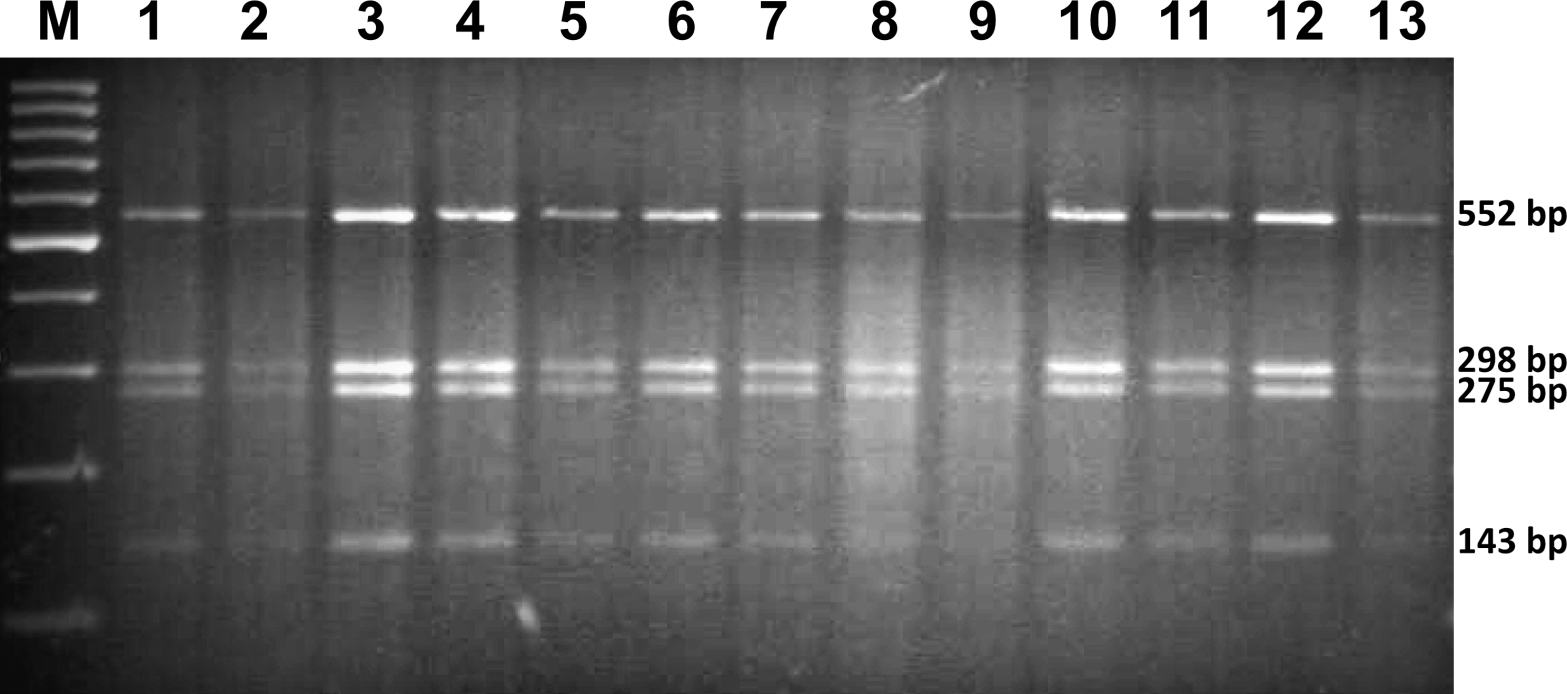
The restriction pattern of *G. salaris*. Lines: 1–5, Kaitum; 6, Juktån; 7–13, Sölvbacka strömmar.** Molecular marker used: ThermoScientific, GeneRuler 100 bp (Waltham, MA, USA).

All 13 analysed specimens were identified as *G. salaris* by means of morphological and molecular methods*.* The obtained PCR-RFLP restriction pattern consisted of four predicted fragments: 552, 298, 275 and 143 bp (Fig. 2). Sequencing showed no differences within the ITS rDNA region. The sequences were deposited in GenBank under accession numbers MG256565, MG256566 and MG256567 (Table 1).

### Phylogenetic analysis based on ADNAM1 sequences

**Figure 3:**
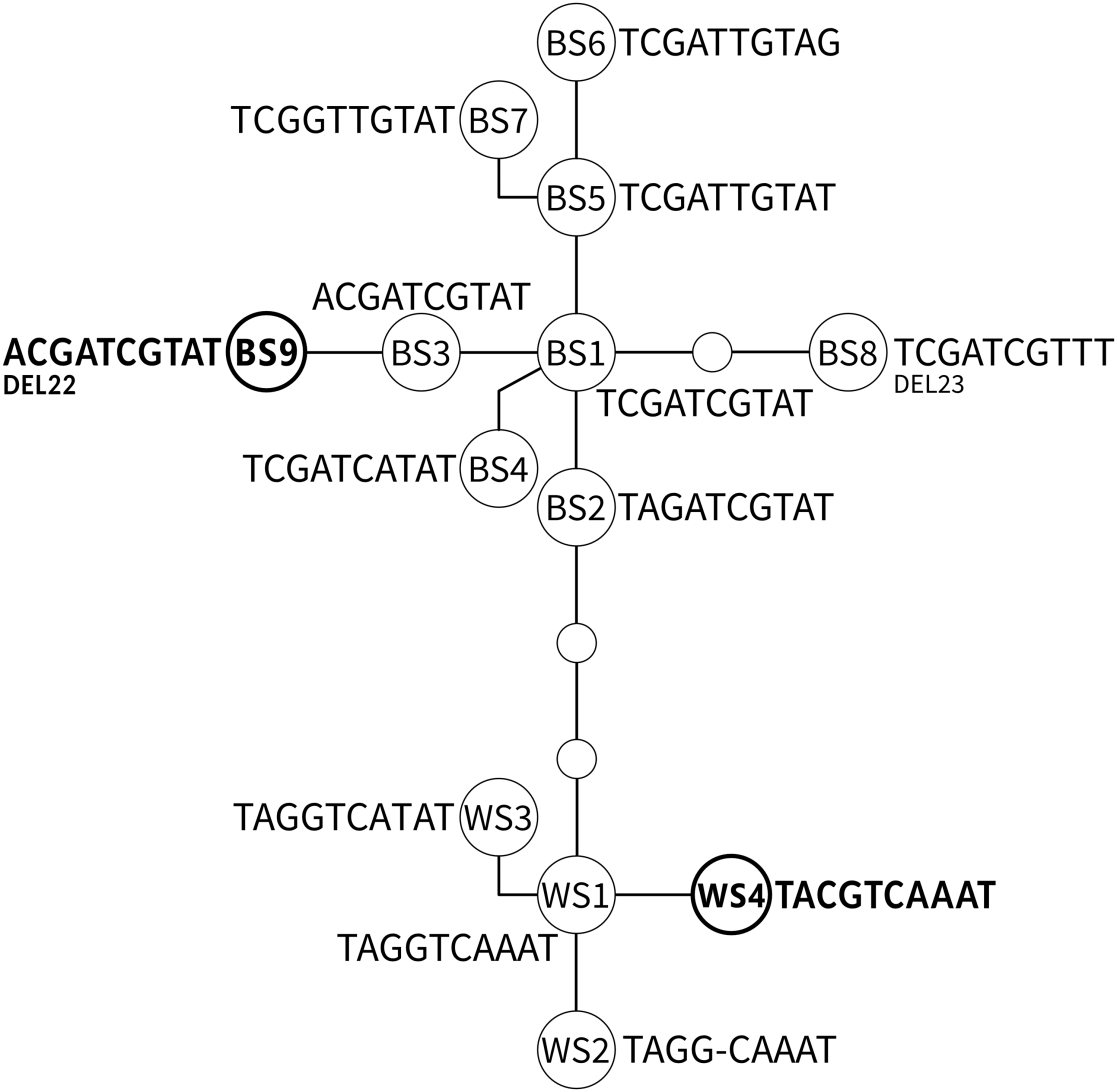
The parsimonious network of the ADNAM1 (Anonymous DNA Marker 1) alleles of *G. salaris*. Newly added alleles are presented in bold. Bent lines indicate the alleles described by Kuusela, Ziętara & Lumme (2007) as nucleotide convertants. Only variable nucleotides are shown. Abbreviations used: BS, allele derived from Baltic Sea; WS, alleles derived from White Sea; DEL, deletion.

A network including all known ADNAM1 alleles is shown in Fig. 3 and listed in Table 2. Two ADNAM1 genotypes were observed in the northernmost location—River Kaitum. The first one, found in three specimens, was a homozygous BS1/BS1 (TCGATCGTAT)—T3 (MG273449). The second one, found in two specimens, was a homozygous BS9/BS9—T6 (MG273450). The BS9 allele stems from the known BS3 allele (ACGATCGTAT), and differs from it by a 22-nucleotide deletion. It has not been reported earlier. Both clones were found on a single fish, however, no BS1/BS9 heterozygotes were detected, therefore no indication of sexual reproduction has been confirmed.

In Juktån, the locality south-west of Kaitum, only a typical and common homozygous T3 genotype (BS1/BS1) was found (MG273451).

The most unexpected genotype was found in all specimens from Sölvbacka (Table 1). They proved to be heterozygous and consist of two alleles—BS1 (TCGATCGTAT, MG273453) from the Baltic Sea watershed and WS4 (TACGTCAAAT, MG273454) from the White Sea watershed. This genotype (TMSRTCRWAT, MG273452) called T7 is very similar to already known S1 (TMGRTCRWAT), yet it includes a diagnostic S (G/C)—a new variable site within ADNAM1 which we here compare and list along the nine described in literature (Kuusela, Ziętara & Lumme, 2007). After conducting molecular cloning it has been determined that the mutation-derived C originates from the alleles occurring in the White Sea watershed (Fig. 3). No recombination was observed which leads to the conclusion that they were clones that came from asexual reproduction. The genotype in question is unique and has never been reported before, more so on grayling.

### Molecular clock hypothesis

The results from the molecular clock calculations revealed the mean substitution rate among branches of 3.6% (95% HPD: 2.2% –5.2%) per million years which corresponds to the divergence rate of 7.2% per million years.

**Figure 4:**
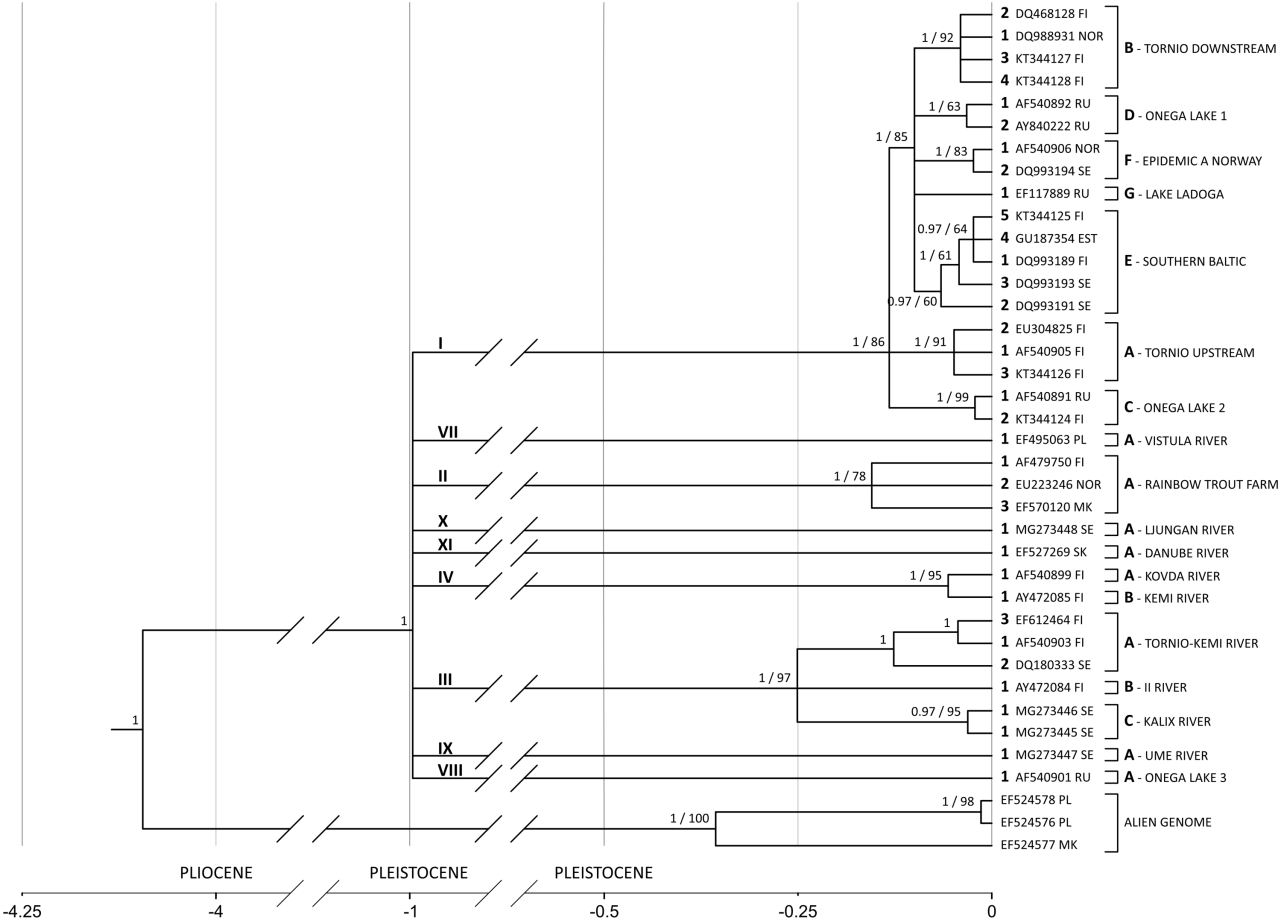
*G. salaris* phylogeny and molecular clock hypothesis based on the complete *cox*1 gene. Numbers at the nodes correspond to the posterior probability (with the cut-off value of 0.97) and bootstrap support, respectively. In case of a node not appearing in one of the analyses, the value is missing. Main lineages and strains are shown with proposed unified nomenclature. The strain code consists of: a Roman numeral designating the lineage (shown above the main lineages), a letter designating the haplotype (shown next to a leaf bracket and before the locality), an Arabic numeral designating the haplotype number (shown preceding an accession number) and a host abbreviation (optional; not included in this phylogeny). Abbreviations: EST, Estonia; FI, Finland; MK, Macedonia; NOR, Norway; PL, Poland; RU, Russia; SE, Sweden; SK, Slovakia; PLIOCENE, 5.33 –2.58 mln; PLEISTOCENE, 2.58 –0.01 mln.

Substitution rate increases along the branch leading from the common ancestor of all salmon-specific strains (lineage I) to the most recent common ancestor (MRCA) of all lineages. If the substitution rate is estimated in the node of MRCA, which results in the highest possible overestimation, it equals *circa* 11% (95% HPD 6.8%–15.8%) leading to the highest divergence rate of 22% per million years (see Fig. 4).

**Table 3:** *Cox1* haplotypes of *G. salaris* considered in present phylogeny.

Accession No.	Hansen, Bachmann & Bakke, 2003/ Hansen, Bakke & Bachmann, 2007		Meinilä et al., 2004/ Kuusela, Ziętara & Lumme, 2007		Isolate	Lineage-strain
	Lineage	Synonym	Synonym	Haplotype		
AF540905^a^	I	–	I	SalBA04	Lataseno, FIN	Tornio upstream **I-A1ss**
EU304825^g^	I	–	I	SalBa12	Lataseno Patoniva, FIN	Tornio upstream, **I-A2ss**
KT344126^i^	I	–	I	SalBa04_487Y	Muonio, FIN	Tornio upstream **I-A3ss**
DQ988931^c^	I	I-B	I	–	Signaldalselva, N	Tornio downstream **I-B1ss**
DQ468128^d^	I	–	I	SalBa05	Turtola, FIN	Tornio downstream **I-B2ss**
KT344127^i^	I	–	I	SalBa05-582YC	Tornio, FIN	Tornio downstream **I-B3ss**
KT344128^i^	I	–	I	SalBa05-1257C	Muonio, FIN	Tornio downstream **I-B4ss**
AF540891^a^AY840223^d^	I	–	I	SalBa1	Morskoy, Kumsa, RUS	Onega Lake 2 **I-C1ss**
KT344124^i^	I	–	I	–	Suna, RUS	Onega Lake 2 **I-C2ss**
AF540892^a^	I	–	I	SalBa02	Sukhoy, RUS	Onega Lake 1 **I-D1ss**
AY840222^d^	I	–	I	SalBa03	Lizhma, RUS	Onega Lake 1 **I-D2ss**
DQ993189^d^	I	–	I	SalBa06	Iijoki, FIN	Southern Baltic, **I-E1ss**
DQ993192^d^DQ993191^d^	I	I-C	I	SalBa08	Genevadsan, Stensan, SE	Southern Baltic, **I-E2ss**
DQ993193^d^	I	–	I	SalBa09	Genevadsan, SE	Southern Baltic, **I-E3ss**
GU187354^h^	I	–	I	SalBa14	Kunda, EST	Soutern Baltic, **I-E4ss**
KT344125^i^	I	–	I	–	Ossaus (Kemijoki), FIN	Southern Baltic, **I-E5ss**
AF540906^a^	I	I-A	I	SalBa07	Vefsna, N	Epidemic A Norway, **I-F1ss**
DQ993194^d^	I	–	I	SalBa10	Hogvadsan, SE	Epidemic A Norway, **I-F2ss**
EF117889^d^	I	–	I	SalBa11	Syskynjoki, RUS	Lake Ladoga **I-G1ss**
AF479750^a^	III	III-F	II	RBT1	FIN	Rainbow trout farm **II-A1om**
DQ517533^b^DQ778628^b^	III	III-F	II	RBT1	Kurzhma, Pista, RUS	Rainbow trout farm **II-A1ss**
EU223246^f^	III	–	II	RBT3	Laerdalselva, N	Rainbow trout farm **II-A2ss**
EF570120^d^	III	–	II	RBT2	Vardar fish farm, MK	Rainbow trout farm **II-A3sl**
AF540903^a^	–	–	III	ThyBa06	Poroeno, FIN	Tornio-Kemi River **III-A1tt**
DQ180333^d^	–	–	III	ThyBa09	Nagereatnu, SE	Tornio-Kemi River) **III-A2tt**
EF612464^d^	–	–	III	ThyBa11	Ounasjoki, FIN	Tornio-Kemi River **III-A3tt**
AY472084^a^	–	–	III	ThyBa08	Soivio, FIN	Iijoki (Ii River) **III-B1tt**
MG273445^k^	–	–	III	–	Kaitum, SE	Kalixälven (Kalix River) **III-C1tt**
MG273446^k^	–	–	III	–	Kaitum, SE	Kalixälven (Kalix River) **III-C1tt**_**ht**_**(**hetorogenic)
AF540899^a^DQ993195^d^	–	-	IV	ThyWs03	Pikkuköngäs, Aventojoki, FIN	Kovda River **IV-A1tt**
AY472085^a^	–	–	IV	ThyWs05	Penninki, FIN	Kem’ River **IV-B1tt**
EF495063^d^	–	T	–	ThyBa10	Radunia, PL	Vistula River **VII-A1tt**
AF540901^a^	–	–	III	ThyBa07	Pyal’ma, RUS	Lake Onega **VIII-A1tt**
MG273447^k^	–	–	–	–	Juktån, SE	Umeälven (Ume River) **IX-A1tt**
MG273448^k^	–	W	–	–	Sölvbacka, SE	Ljungan River **X-A1tt**
EF527269^e^	VI	N	–	–	Hnilec, SK	Danube River **XI-A1tt**

**Notes.**

a Meinilä et al., 2004

b Ziętara, Kuusela & Lumme, 2006

c Huyse et al., 2007

d Kuusela, Ziętara & Lumme, 2007

e Plaisance et al., 2007

f Ziętara, Johnsen & Lumme, 2008

g Kuusela et al., 2009

h Ozerov et al., 2010

i Lumme et al., 2016

k Present study

Abbreviations used EST Estonia FIN Finland MK Macedonia N Norway PL Poland RUS Russia SE Sweden SK Slovakia ss *Salmo salar* sl *Salmo lentica* om *Oncorhynchus mykiss* tt *Thymallus thymallus*

Strain code consists of: a Roman numeral designating the lineage, a letter designating the haplotype, an Arabic numeral designating the haplotype number and a host abbreviation (optional).

### Phylogenetic analysis based on *cox*1 sequences

A phylogenetic hypothesis based on the complete *cox*1 gene (1,548 bp) is shown in Fig. 4. The tree is starlike and consists of nine equidistant lineages with MRCA estimated to live in the Pleistocene around 1 Mya (95% HPD: 1.84–0.49). On the basis of the phylogeny presented on Fig. 4, a new nomenclature unifying previous systems is introduced (Table 3). It consists of: (1) a Roman numeral designating the lineage, (2) a letter designating the haplotype, (3) an Arabic numeral designating the haplotype number and (4) a host abbreviation (optional). For example, label II-A1_ss_ designates a parasite carrying haplotype 1, which infects *Salmo salar* and represents the strain A (rainbow trout farm) of the lineage II. II-A1_om_ designates a parasite with the haplotype 1 from the lineage II and the strain A but infecting *Oncorhynchus mykiss*.

The lineages presented are as follows. The salmon-specific lineage I, which includes seven salmon-specific strains that are spatially distributed and well supported in our analysis. It originated in the Baltic basin but recently transferred to the basins of the White, Norwegian and North seas, being responsible for Norwegian and Karelian salmon epidemics.

Rainbow trout-specific lineage II (RBT) present mainly in northern European fish hatcheries consists of two strains—one strictly connected to rainbow trout industry (II-A1) and the other living in southern Europe (II-B1sl). The strain specific to rainbow trout that switched to salmon consists of two haplotypes (II-A1 and II-A2) that differ by only one nucleotide (dGTR+Γ+I = 0.00064, Table S1) which points to the divergence time of about 38,000 years ago. It means that two clones managed to switch to rainbow trout after its introduction to Europe.

The lineage III is reported in the Baltic Sea basin rivers. Although it had consisted of several haplotypes earlier (mostly from Finland), it has been further extended in this study. The strains III-A (Kemijoki and Tornionjoki) present in Kemi and Tornio Rivers as well as strain III-B (Iijoki) from river Ii have reached high level of node support. A new strain III-C from Sweden has been reported in present study in Kalix river (Kalixälven) system, extending the lineage distribution. Two haplotypes, called III-C1tt and III-C1tt_ht_, were found on a single grayling which is extremely rare considering the competing nature of *G. salaris* clones. III-C1tt haplotypes form a separate strain within the lineage typical for *G. salaris* living on grayling in the Baltic Sea basin. III-C1tt appears in two forms–homogenic (AGGGCT, MG273445; three specimens found) or heterogenic (RRSRYY, MG273446; two specimens found) (Table 1). They bear most resemblance to the Ounasjoki III-A3tt (ThyBa11) (EF612464) haplotype, with the calculated General Time Reversible *d*_GTR+Γ+*I*_ distances of 0.005 (homozygous form) and 0.002 (heterozygous form) (Fig. 4, Table S1). Additionally, these two forms of III-C1tt differ also in ADNAM1. Two further strains have shown within the lineage typical for *G. salaris* living on grayling in the Baltic Sea basin—one from Ii River (Iijoki, AY472084) and the other from Kemi (Kemijoki, EF612464) and Tornio (Tornionjoki, AF540903, DQ180333) Rivers systems. All three strains of lineage III–Tornionjoki (III-A), Iijoki (III-B) and Kalixalven (III-C) diverged from a common ancestor about 300,000 years ago. All six heterogenic nucleotides are a combination of III-C1tt (AGGGCT) haplotype present in the locality and GACATC observed elsewhere (except those reported in Poroeno–AF540903, Soivio –AY472084, Nagereatnu–DQ180333, Radunia–EF495063 and Ounasjoki–EF612464). Locus 1485 seems to be a hot spot as it has been proven to contain T, C and G nucleotides in other Baltic isolates. These observations may not only reveal a transient state of mitochondrial genome but also suggest biparental inheritance of mitochondria, however, such phenomenon has never been reported before for Platyhelminthes.

The lineage IV reported in the White Sea basin rivers. It is represented by two strains: IV-A from Kovda (Kouda) River system, including haplotypes IV-A1 from Pikkuköngäs and Aventojoki, and the other IV-B1 from Kem’ (Kemi) River system (Penninki haplotype (Bayesian posterior probability equals 1 and bootstrap support 95, respectively).

The lineage VII specific to grayling is reported from Poland. It consists of a single haplotypes A1 observed in river Radunia from Wisła river (VII-A1tt) river system. There is also another haplotype in GenBank (DQ159922) reported from Brda, the tributary of Wisła (Table 3). The haplotype differs from the one from the Radunia haplotype (EF495063) by only six transitions and two transversions (dGTR+Γ+I = 0.01, Table S1), therefore both haplotypes may belong to the lineage from Wisła (Fig. 4). The sequence of haplotype from Brda was short and thus excluded from the present analysis.

The lineage VIII is created by the Pyal’ma (Pälmä, AF540901) river haplotype A1 from lake Onega basin.

Haplotype IX-A1tt (MG273447) found in present study in Juktån (Ume river drainage system) forms a separate lineage IX within the phylogeny. The calculated GTR+Γ+I distance was also the smallest for the Ounasjoki sequence (III-A3tt [ThyBa11], EF612464). It equalled 0.012 which is much higher than the values calculated for the Kaitum haplotypes (Table S1). The addition of IX-A1tt to the phylogenetic tree changes the previously reported mitochondrial haplotype relationships within the Kalixalven, Iijoki and Tornio strains. Being a main lineage, IX-A1tt removes the Russian Pyal’ma VIII-A1tt (ThyBa07, AF540901) haplotype from the most basal position of the clade and separating it as yet another main lineage–diverged radially about 1 Mya in the same refugium as the other, more successful lineage. However, sample bias cannot be excluded (Fig. 4).

The most unusual of all newly found haplotypes is X-A1tt (MG273448) observed in Sölvbacka strömmar (Ljungan river drainage system). It forms a completely new lineage equally distant from all of eight deep lineages shown in Fig. 4 (mean *d*_GTR+Γ+*I*_ equals 0.022, Table S1). There is a possibility of it belonging to the same lineage as the Murusjøen sequence (W, DQ159928) as they differ by only four T/C transitions (*d*_GTR+Γ+*I*_ = 0.005, Table S1). However, the W haplotype is only 782 bp long which is why it does not contribute to this phylogenetic analysis. Moreover, specimens of this haplotype proved to have heterozygous ADNAM1 consisting of alleles from Baltic and White Sea watersheds.

The lineage XI reported from Danube (XI-A1tt) river system. The Danubian lineage used to be officially described as *G. thymalli* (EF527269), however, it has been recently synonymised with *G. salaris* which is also supported by present phylogeny (Fig. 4).

## Discussion

### Shortcomings of *G. salaris* phylogeny

Although *G. salaris* mitochondrial phylogeny has been studied extensively for more than a decade (Meinilä et al., 2002) it is far from being complete. Its phylogeography and evolution still pose a challenge for researchers which is especially true for parasites living on grayling. The most comprehensive phylogenetic analysis to date showed that adding as few as six new grayling-specific *cox*1 haplotypes (from England, Poland and Norway) extended the phylogenetic tree by as many as five main lineages (Hansen, Bakke & Bachmann, 2007). Their research combined 44 *cox*1 haplotypes from previous studies done by Hansen, Bachmann & Bakke (2003), Hansen et al. (2006), Meinilä et al. (2004) and Robertsen et al. (2007). The resulting tree supported prior knowledge about the polytypic structure of *G. salaris*. It is worth noticing that in all probability, the tree included more than 10 main mitochondrial lineages contrary to its proposed topology. The fact derived from insufficient length of some of the sequences (745 bp) leading to poor support of the main lineages. Therefore, earlier attempt at integrating differing nomenclatures existing at the time proposed by Hansen, Bachmann & Bakke (2003) and Hansen, Bakke & Bachmann (2007) and Meinilä et al. (2004) was not successful. The problem with statistical support was solved to some extent by Kuusela, Ziętara & Lumme (2007) analysing a 1,600 bp fragment (including a complete *cox*1 gene sequences). Unfortunately, many main lineages were not represented since they only comprised short sequences.

Moreover, all previous research struggled with the status of *G. thymalli*. In 2003, Ziętara & Lumme (2003) suggested its conspecificity with *G. salaris*. The idea was based on the phylogeny of *Gyrodactylus Limnonephrotus* as well as the accuracy of the ITS rDNA species identification. Hansen, Bachmann & Bakke (2003), in turn, based their theory on the host specificity of the parasites assigning all specimens from grayling to *G. thymalli* (Latvia, Norway and Sweden) and the remaining ones as *G. salaris*. However, such approach led to the conclusion that the species might represent one, two or more species due to lack of monophyly of the haplotypes. Meinilä et al. (2004) ascribed all of the studied strains from Northern Europe to *G. salaris*, leaving the question of *G. thymalli* unanswered. The first attempt to synonymise the species stimulated the research to support (Sterud et al., 2002; Cunningham et al., 2003; Shinn et al., 2004; Hansen, Bakke & Bachmann, 2007) or reject (Hansen et al., 2006; Ziętara et al., 2010) the species rank of *G. thymalli*. The conspecificity theory has recently been supported thus synonymising the species (Fromm et al., 2014). The salmon specific lineage has been recently revised by Lumme et al. (2016), while other lineages have not been discussed. Therefore, a thorough revision of *G. salaris* phylogeny was necessary for correct management of gyrodactylosis in the affected countries.

It is now clear that phylogeny based on the complete *cox*1 gene sequence offers better phylogenetic signal and provides a more correct phylogeny when combined with the inheritance data derived from nuclear genome analysis. It finds proof in other analyses such as studies on: gyrodactylosis in Estonia (Ozerov et al., 2010), introgression of alien mitochondrial genome into *G*. *salaris* (Ziętara et al., 2010), salmon-specific lineage of *G*. *salaris* (Lumme et al., 2016), and Eurasian minnow *Phoxinus phoxinus* (L.) *Gyrodactylus* spp. phylogeny (Lumme, Ziętara & Lebedeva, 2017), as well as the phylogeny presented in this research.

### Phylogeny of *G. salaris* based on mitochondrial *cox*1 sequences

The phylogeny presented in this study comprises nine equidistant lineages and the lineage reported by Lindenstrm et al. (2003).

The salmon-specific lineage I, described formally by Malmberg (1957) as *G. salaris* which includes seven salmon-specific strains originating in the Baltic basin was recently transferred to the basins of the White, Norwegian and North seas (Hansen, Bachmann & Bakke, 2003; Meinilä et al., 2004).

The lineage II is common in rainbow trout hatcheries in Finland, Sweden, Denmark, Norway, Russia and Italy (Hansen, Bachmann & Bakke, 2003; Meinilä et al., 2004; Kania, Jørgensen & Buchmann, 2007; Paladini et al., 2009; Ziętara, Kuusela & Lumme, 2006; Ziętara, Johnsen & Lumme, 2008). Interestingly, the lineage has not been observed in Poland yet (Rokicka, Lumme & Ziętara, 2007). It was also reported on Arctic charr *Salvelinus alpinus* (L.) in Norway (Olstad et al., 2007; Robertsen et al., 2007). Although a number of haplotypes has been reported in Italy, none of them have been deposited in GenBank (Paladini et al., 2009). The lineage was also found on Ohrid trout *Salmo letnica* (Karaman, 1924) in the Mediterranean region (Ziętara et al., 2010), from where the lineage may have actually originated. It is most probable that it was introduced to northern Europe via rainbow trout industry.

The lineage III extended in this study was previously reported from river Ii (Iijoki), river Kemi (Kemijoki) and river Tornio (Tornionjoki) (Meinilä et al., 2004 and Kuusela, Ziętara & Lumme, 2007).

The lineage IV represented by two strains was reported in the White Sea basin from Kovda (Kouda) River sytem, including Pikkukongas (Meinilä et al., 2004) and Aventojoki (Kuusela, Ziętara & Lumme, 2007), and from Kem’ (Kemi) River system (Penninki haplotype, Meinilä et al., 2004).

The lineage VII was reported from Poland in river Radunia (Kuusela, Ziętara & Lumme, 2007) and possibly in river Brda (T–Hansen, Bakke & Bachmann, 2007), both from the Wisła river basin.

The lineage VIII created by the Pyal’ma (Pälmä) river haplotype from lake Onega basin was reported earlier in lineage III (Meinilä et al., 2004). In present phylogeny it has been separated due to the introduction of the Ume river haplotype.

There are two new lineages, IX and X, formed by single haplotypes: from Juktån (Ume river drainage system) and from Sölvbacka strömmar (Ljungan river drainage system). It is worth mentioning, however, that the latter clusters with the W hyplotype (DQ159928 by Hansen, Bakke & Bachmann, 2007) suggesting that the lineage may also include this haplotype.

The lineage XI reported in the Danube River system (N in Hansen, Bakke & Bachmann, 2007, EF527269 by Plaisance et al., 2007). Five of the sequences (DQ159914, DQ159915, DQ159916, DQ159917, AY486551) from this locality deposited in GenBank seemed to represent the original *cox*1 haplotype XI-A1 of the type specimen for *G. thymalli*, as they were identical within their length. Still, they were short and neither ITS rDNA nor ADNAM1 marker have been reported for this lineage. Furthermore, *G. thymalli* originally described by Žitňan (1960) has recently been synonymised with *G. salaris* (Fromm et al., 2014)*.*

Additionally, there exists the lineage reported originally by Lindenstrm et al. (2003), the only one of different distance within this set, found on rainbow trout in Denmark and later in Poland (Rokicka, Lumme & Ziętara, 2007). Its more extensive molecular analysis has suggested it to stem from hybridisation between *G. salaris* and an unknown *Gyrodactylus* sp. resulting in alien mitochondria introgression (Rokicka, Lumme & Ziętara, 2007; Ziętara et al., 2010).

At least five more main lineages of this polytypic species have been known to date (Hansen, Bachmann & Bakke, 2003; Hansen et al., 2006; Hansen, Bakke & Bachmann, 2007; Meinilä et al., 2004; Kuusela, Ziętara & Lumme, 2007; Ziętara et al., 2010) and even more are expected to appear when the host distribution coverage improves. The five lineages are not represented in this research due to lack of complete *cox*1 and/or ADNAM1 sequences but they are considered in the unified nomenclature presented in this research. Two of them (V and VI) were reported from Norway (Hansen, Bachmann & Bakke, 2003; Hansen et al., 2006; Hansen, Bakke & Bachmann, 2007). Lineage V consisting of eight haplotypes, was found in Glomma River system. The river together with its tributaries (Åsta, Gudbrandsdalslågen and Rena) and lakes (Mjøsa and Lesjaskogsvatnet) drains directly into the Oslo fjord–part of Skagerrak strait, which connects the North Sea with Kattegat. Lineage VI consisting of three haplotypes, was found in Trysilelva River of the Baltic Sea basin which flows from Norway to the Swedish lake Vänern. Each of the remaining three lineages (XII, XIII and XIV) is represented only by a single haplotype. Lineage XII (V–DQ159924 –DQ154925 in Hansen, Bakke & Bachmann, 2007) was found in river Test (United Kingdom), lineage XIII (U–DQ159923 in Hansen, Bakke & Bachmann, 2007)–in river Gwda (Odra river system, Poland) and lineage XIV (GQ370816, Paladini et al., 2009) was collected in a rainbow trout fish farm on river Nera in Italy—a tributary of Tiber (Tevere) river which flows into the Tyrrhenian Sea. The haplotype is related to haplotypes found in Romania (GQ129460, GQ129461, GQ129462 and GQ129463) and both pseudogenes reported earlier (AY225307 –AY225308).

Although *G. salaris* phylogeny constructed on the basis of a single mitochondrial *cox*1 marker and supplemented by information derived from two additional nuclear markers (ITS rDNA and ADNAM1) seems to describe this hemiclonal species complex genetic structure well, the mechanism leading to such a structure remains elusive. The new genomic approach (Hahn, Fromm & Bachmann, 2014) opens unlimited possibilities to study the mechanism shaping the genetic diversity of this dangerous yet intriguing species.

### Evidence for Eemian crossing between lineages living on grayling

Concerning evolution, the most interesting lineage researched herein has been the one found in the Ljungan river drainage system (lineage X). It has heterozygous ADNAM1 consisting of alleles originating in the Baltic and the White Sea basins. Such combination of alleles has been demonstrated to be typical exclusively for the salmon-specific and RBT lineages (I and II, respectively).

It has been explained by Kuusela, Ziętara & Lumme (2007) that the Baltic and White Sea lineages specific for grayling crossed in the Eemian interglacial, switched host, and gave rise to the salmon-specific lineage I. For such a scenario to have occurred, two steps would have had to take place: the crossing between lineages and the host-switch. The haplotype found in Ljungan river system clearly confirms the first step as it proves the clone offspring of the original hybrids to still live on grayling today.

As has been shown by Kuusela, Ziętara & Lumme (2007), presence of the WS/BS heterozygosity is a necessary requirement for correct host recognition making sexual reproduction a serious risk from the evolutionary standpoint. How precise the recognition must be has been demonstrated recently by Lumme et al. (2016) as a result of a ten-year study. For its duration a genetically variable *G. salaris* population living in the 522 km long Tornio River, first reported as spatially differentiated and not panmictic by Kuusela et al. (2009), was shown to remain strongly and stably genetically differentiated among the upper and lower river nurseries in spite of annual flux of hosts. The genetic structure was consistent with significant spatial differentiation of salmon, suggesting local co-adaptation of the host-parasite subpopulations. The parasite reproduced mostly asexually preventing gene segregation, which would be disadvantageous for maintaining the co-adaptation.

If such fine mechanism of host-parasite interaction is true for salmon-specific strains, it should also be observed for the grayling-specific ones. Indeed, all seven specimens collected from wild graylings in the Ljungan river system as part of this research were heterozygous and therefore confirming the preference of asexual reproduction.

### Molecular clock hypothesis

The most important salmon-specific lineage—lineage I, described formally by Malmberg (1970) as *G. salaris*, includes parasites currently living on salmon in the basins of the White, Norwegian, Baltic and North seas (Hansen, Bachmann & Bakke, 2003; Meinilä et al., 2004). What is worth noticing, it accommodates the strain (I–F) responsible for killing the juvenile Atlantic salmons in the past epidemics (Johnsen & Jensen, 1991; Johnsen & Jensen, 1992; Kudersky, Ieshko & Schulman, 2003). The lineage is rather diverse and quite well characterized. It consists of seven strains including many haplotypes (Kuusela, Ziętara & Lumme, 2007; Lumme et al., 2016). The lineage was explained to have originated from hybridization of two grayling-specific strains during a relatively short time—less than 3,000 years of a connection existing between the White and Baltic seas during the early Eemian interglacial period about 132,000–130,000 years ago (Funder, Demidov & Yelovicheva, 2002). This molecular clock hypothesis was recently challenged by Hahn et al. (2015). The authors used another indirect calibration applying the divergence time of ∼0.6 My between the Atlantic and Danubian *Salmo trutta* Linnaeus, 1758 lineages to the corresponding node in the *Gyrodactylus teuchis* Lautraite, Blanc, Thiery, Daniel & Vigneulle, 1999 phylogeny, which was based on *cox*1 mitochondrial haplotypes. They inferred a mean substitution rate of 5.1% (95% HPD 2.9–7.7%) per million years which leads to the divergence rate of about 10.2% (Wilke, Schulthei & Albrecht, 2009).

The divergence rate between the lineages of 13.7–20.3% per million years was calculated by Meinilä et al. (2004) based on the mean divergence between them. Kuusela, Ziętara & Lumme (2007) further adjusted the calculations by utilizing the Kimura’s 2-parameter distances, and obtaining the divergence rate values between 13.1% and 7.6% (for the Göta haplotype and Onega 2 strain, respectively). The endemic Göta haplotype was reported for landlocked salmon populations from the river Göta—a relic from the Narke strait, which was the first Atlantic connection of recovering Baltic Sea about 10 000 years ago. Nowadays, the river drains lake Vänern into the Kattegat in Göteborg. The area is considered to be a part of the western edge of the Baltic Sea basin (Nilsson et al., 2001). The Göta haplotype and its most basal position is crucial for the molecular time estimation. In the phylogeny reported by Hansen, Bachmann & Bakke (2003) and Hansen, Bakke & Bachmann (2007), it was excluded from salmon-specific lineage I, on which the calculations were based, however, it remained a part of the clade in the phylogeny reported by Meinilä et al. (2004). Although the haplotype was removed from the phylogeny reported by Kuusela, Ziętara & Lumme (2007), its node was used for the calculation of the upper limit divergence rate (13.1%). It led to the mean substitution rate of about 6.6%, which was above the rate of 5.1% given by Hahn et al. (2015).

In this study the calculations were further adjusted. The best-fit evolution model for the *cox*1 gene was utilised. It resulted in obtaining the following mean substitution and divergence rates: the lowest underestimate for the common ancestor of all salmon-specific haplotypes (lineage I)—3.6% and 7.2% per million years, respectively; the highest overestimate for the node of MRCA—11% and 22% per million years, respectively. Clearly, the substitution rate and divergence rate values obtained in 2015 by Hahn et al. (5.1% and 10.2%, respectively) also fall within this range (Hahn et al., 2015). Applying the estimates (3.6% and 5.1%), the divergence times of MRCA equal 1 and 0.7 Mya for *G. salaris* lineages*,* respectively, placing the MRCA within Pleistocene, which is in accordance with the grayling *T. thymallus* lineages evolution (Bernatchez, 2001; Gum, Gross & Kuehn, 2005).

The more data sets are analysed, the more clearly the fascinating history of *G. salaris* evolution can be understood. Therefore, there remains much to explore—new lineages are still expected to be found as to this moment the research in the field has focused mainly on the northern and central parts of Europe, yet the wageneri group has been known to far exceed this area (Lumme, Ziętara & Lebedeva, 2017) which is also true for the species of grayling.

## Conclusions

Our study revealed a second case of ancient crossing between the Baltic and White Sea lineages of *G. salaris*, which happened about 130,000 years ago in the connection between the two seas. Surprisingly, the resulting strain survived on grayling and is living in central Sweden.

Robust phylogenetic analyses of the complete *cox*1 gene enabled the adjusting of the molecular clock estimates, resulting in obtaining the mean divergence rate of 7.2% per million years.

Based on the revised *G. salaris* phylogeny as well as the molecular clock hypothesis, we have proposed a unified nomenclature for the parasite lineages and strains facilitating accurate management of gyrodactylosis.

##  Supplemental Information

10.7717/peerj.5167/supp-1 Table S1 Distance matrix Maximum-likelihood distance matrix (GTR+G+I model) created in PAUP 4.0b10 (Swofford, 2002).

10.7717/peerj.5167/supp-2 Supplemental Information 1 *cox1* BEAST input file

10.7717/peerj.5167/supp-3 Supplemental Information 2 *Cox1* matrix used in the study

10.7717/peerj.5167/supp-4 Supplemental Information 3 Sequences obtained The file contains all of the obtained sequences in three FASTA files. Every FASTA file is named as the marker it includes.

## Additional Information and Declarations

### DNA Deposition

The following information was supplied regarding the deposition of DNA sequences: The ITS sequences described here are accessible via GenBank under accession numbers MG256565, MG256566 and MG256567. The *cox*1 sequences described here are accessible via GenBank under accession numbers MG273445, MG273446, MG273447 and MG273448. The ADNAM1 sequences described here are accessible under accession numbers MG273449 to MG273454.

### Data Availability

The following information was supplied regarding data availability: The raw data are provided in the Supplemental Files.
