# Supplementary material for: Revision of Gyrodactylus salaris phylogeny inspired by new evidence for Eemian crossing between lineages living on grayling in Baltic and White sea basins
Source: PeerJ. 2018 Jul 6;6:e5167. doi: 10.7717/peerj.5167 (PMC6077759; doi:10.7717/peerj.5167)
Supplement: Table S1 — Maximum-likelihood distance matrix (GTR+G+I model) created in PAUP 4.0b10 (Swofford, 2002). [file peerj-06-5167-s001.pdf]

Maximum-likelihood distance matrix

Likelihood settings:

Number of substitution types = 6

User-specified substitution rate matrix =

-	2.259900	6.588600	0.908300
2.259900	-	0.834000	12.343600
6.588600	0.834000	-	1.000000
0.908300	12.343600	1.000000	-

Assumed nucleotide frequencies (set by user):

A=0.26460 C=0.19980 G=0.20720 T=0.32840

Among-site rate variation:

Assumed proportion of invariable sites = 0.5165

Distribution of rates at variable sites = gamma (continuous) with shape parameter (alpha) = 0.6865

These settings correspond to the GTR+G+I model

		1	2	3	4	5	6	7
1	sal DQ468128 Sal	-						
2	sal DQ988931 Sig	0.00065	-					
3	sal KT344127 Sal	0.00064	0.00130	-				
4	sal KT344128 Sal	0.00065	0.00130	0.00129	-			
5	sal AF540892 Sal	0.00458	0.00527	0.00524	0.00525	-		
6	sal AY840222 Sal	0.00325	0.00393	0.00391	0.00392	0.00129	-	
7	sal AF540906 Sal	0.00461	0.00531	0.00528	0.00529	0.00394	0.00394	-
8	sal DQ993194 Sal	0.00394	0.00463	0.00460	0.00461	0.00327	0.00327	0.00065
9	sal EF117889 Sal	0.00524	0.00594	0.00591	0.00591	0.00457	0.00457	0.00461
10	sal KT344125 Oss	0.01150	0.01227	0.01220	0.01222	0.01079	0.01079	0.01088
11	sal GU187354 Sal	0.00526	0.00596	0.00592	0.00593	0.00458	0.00458	0.00462
12	sal DQ993189 Sal	0.00526	0.00596	0.00593	0.00593	0.00459	0.00458	0.00462
13	sal DQ993193 Sal	0.00393	0.00462	0.00459	0.00460	0.00326	0.00326	0.00329
14	sal DQ993191 Sal	0.00460	0.00529	0.00526	0.00527	0.00393	0.00393	0.00396
15	sal EU304825 Sal	0.00663	0.00735	0.00730	0.00732	0.00595	0.00594	0.00599
16	sal AF540905 Sal	0.00594	0.00665	0.00662	0.00663	0.00527	0.00527	0.00531
17	sal KT344126 Sal	0.00662	0.00733	0.00729	0.00730	0.00594	0.00593	0.00598
18	sal AF540891 Sal	0.01079	0.01155	0.01148	0.01150	0.01008	0.01008	0.01017
19	sal KT344124 Sun	0.00459	0.00528	0.00526	0.00526	0.00392	0.00392	0.00395
20	thy AF540899 Thy	0.02817	0.02911	0.02894	0.02900	0.02736	0.02735	0.02761
23	thy MG273445 Kai	0.03447	0.03549	0.03527	0.03534	0.03361	0.03360	0.03393
24	thy MG273446 Kai	0.02939	0.03035	0.03017	0.03023	0.02856	0.02855	0.02882
25	thy MG273448 Sol	0.02262	0.02350	0.02337	0.02341	0.02334	0.02333	0.02355
26	thy MG273447 Juk	0.02893	0.02988	0.02971	0.02976	0.02812	0.02811	0.02662
27	let EF570120 far	0.03097	0.03193	0.03175	0.03181	0.03171	0.03170	0.03200
28	myk AF479750 RBT	0.02871	0.02964	0.02947	0.02953	0.02790	0.02789	0.02815
30	sal EU223246 RBT	0.02793	0.02886	0.02869	0.02875	0.02713	0.02712	0.02737
31	thy AF540901 Thy	0.03065	0.03162	0.03144	0.03150	0.02983	0.02982	0.03011
32	thy AF540903 Thy	0.03595	0.03698	0.03676	0.03683	0.03510	0.03508	0.03543
33	thy AY472084 Thy	0.03599	0.03702	0.03680	0.03687	0.03594	0.03512	0.03628
34	thy AY472085 Thy	0.02817	0.02911	0.02894	0.02900	0.02736	0.02735	0.02761
35	thy DQ180333 Thy	0.03348	0.03448	0.03428	0.03435	0.03263	0.03262	0.03294
36	thy EF495063 Thy	0.02940	0.03036	0.03018	0.03024	0.02857	0.02856	0.02884
37	thy EF527269 Hni	0.03607	0.03708	0.03687	0.03621	0.03523	0.03521	0.03556
38	thy EF612464 Thy	0.02752	0.02845	0.02829	0.02834	0.02671	0.02670	0.02695

Maximum-likelihood distance matrix (continued)

	8	9	10	11	12	13	14
8 sal DQ993194 Sal	-						
9 sal EF117889 Sal	0.00393	-					
10 sal KT344125 Oss	0.01016	0.01149	-				
11 sal GU187354 Sal	0.00394	0.00525	0.01019	-			
12 sal DQ993189 Sal	0.00394	0.00525	0.01087	0.00129	-		
13 sal DQ993193 Sal	0.00262	0.00392	0.00948	0.00129	0.00129	-	
14 sal DQ993191 Sal	0.00328	0.00459	0.01084	0.00327	0.00327	0.00195	-
15 sal EU304825 Sal	0.00530	0.00662	0.00941	0.00594	0.00664	0.00529	0.00597
16 sal AF540905 Sal	0.00463	0.00594	0.00871	0.00526	0.00596	0.00462	0.00529
17 sal KT344126 Sal	0.00530	0.00661	0.00940	0.00593	0.00663	0.00528	0.00596
18 sal AF540891 Sal	0.00945	0.01078	0.00065	0.00948	0.01016	0.00877	0.01013
19 sal KT344124 Sun	0.00328	0.00458	0.01017	0.00065	0.00065	0.00065	0.00261
20 thy AF540899 Thy	0.02676	0.02813	0.02423	0.02668	0.02757	0.02601	0.02749
23 thy MG273445 Kai	0.03302	0.03442	0.03100	0.03292	0.03387	0.03224	0.03378
24 thy MG273446 Kai	0.02795	0.02934	0.02605	0.02787	0.02878	0.02720	0.02870
25 thy MG273448 Sol	0.02273	0.02409	0.02097	0.02266	0.02351	0.02201	0.02345
26 thy MG273447 Juk	0.02577	0.02889	0.02498	0.02744	0.02833	0.02678	0.02826
27 let EF570120 far	0.03113	0.03249	0.02954	0.03193	0.03195	0.03038	0.03186
28 myk AF479750 RBT	0.02730	0.02867	0.02641	0.02810	0.02811	0.02657	0.02804
30 sal EU223246 RBT	0.02653	0.02789	0.02564	0.02732	0.02733	0.02579	0.02726
31 thy AF540901 Thy	0.02923	0.03061	0.02732	0.02914	0.03005	0.02848	0.02998
32 thy AF540903 Thy	0.03450	0.03590	0.03247	0.03440	0.03536	0.03373	0.03527
33 thy AY472084 Thy	0.03535	0.03675	0.03330	0.03524	0.03621	0.03457	0.03611
34 thy AY472085 Thy	0.02676	0.02813	0.02264	0.02668	0.02757	0.02601	0.02749
35 thy DQ180333 Thy	0.03203	0.03343	0.03005	0.03194	0.03288	0.03127	0.03279
36 thy EF495063 Thy	0.02796	0.02936	0.02618	0.02856	0.02947	0.02789	0.02871
37 thy EF527269 Hni	0.03465	0.03442	0.03380	0.03474	0.03549	0.03388	0.03540
38 thy EF612464 Thy	0.02610	0.02748	0.02425	0.02602	0.02691	0.02536	0.02684

Maximum-likelihood distance matrix (continued)

	15	16	17	18	19	20	23
15 sal EU304825 Sal	-						
16 sal AF540905 Sal	0.00065	-					
17 sal KT344126 Sal	0.00129	0.00064	-				
18 sal AF540891 Sal	0.00871	0.00801	0.00870	-			
19 sal KT344124 Sun	0.00597	0.00528	0.00595	0.00946	-		
20 thy AF540899 Thy	0.02339	0.02418	0.02494	0.02344	0.02678	-	
23 thy MG273445 Kai	0.03110	0.03026	0.03104	0.03016	0.03305	0.02616	-
24 thy MG273446 Kai	0.02614	0.02532	0.02609	0.02524	0.02798	0.02137	0.00000
25 thy MG273448 Sol	0.02104	0.02026	0.02100	0.02020	0.02275	0.01802	0.02288
26 thy MG273447 Juk	0.02573	0.02493	0.02569	0.02418	0.02754	0.01743	0.01755
27 let EF570120 far	0.02963	0.02880	0.02957	0.02872	0.03115	0.02720	0.03747
28 myk AF479750 RBT	0.02582	0.02501	0.02577	0.02561	0.02733	0.02563	0.03255
30 sal EU223246 RBT	0.02505	0.02425	0.02500	0.02484	0.02655	0.02486	0.03175
31 thy AF540901 Thy	0.02741	0.02660	0.02736	0.02651	0.02926	0.02265	0.02287
32 thy AF540903 Thy	0.03257	0.03172	0.03251	0.03162	0.03453	0.02761	0.00809
33 thy AY472084 Thy	0.03340	0.03255	0.03334	0.03244	0.03538	0.02841	0.00878
34 thy AY472085 Thy	0.02339	0.02418	0.02494	0.02344	0.02678	0.00130	0.02616
35 thy DQ180333 Thy	0.03015	0.02931	0.03009	0.02921	0.03206	0.02525	0.00602
36 thy EF495063 Thy	0.02452	0.02533	0.02610	0.02700	0.02867	0.02547	0.03084
37 thy EF527269 Hni	0.03365	0.03280	0.03358	0.03295	0.03468	0.02983	0.03285
38 thy EF612464 Thy	0.02433	0.02353	0.02429	0.02346	0.02613	0.01966	0.00533

Maximum-likelihood distance matrix (continued)

	24	25	26	27	28	30	31
24 thy MG273446 Kai	-						
25 thy MG273448 Sol	0.01823	-					
26 thy MG273447 Juk	0.01307	0.01729	-				
27 let EF570120 far	0.03233	0.02395	0.02806	-			
28 myk AF479750 RBT	0.02757	0.01947	0.02648	0.00659	-		
30 sal EU223246 RBT	0.02678	0.01874	0.02571	0.00592	0.00064	-	
31 thy AF540901 Thy	0.01899	0.01951	0.01880	0.03201	0.02567	0.02490	-
32 thy AF540903 Thy	0.00463	0.02431	0.01895	0.03732	0.03242	0.03162	0.02591
33 thy AY472084 Thy	0.00531	0.02509	0.01823	0.03654	0.03165	0.03085	0.02516
34 thy AY472085 Thy	0.02137	0.01802	0.01743	0.02720	0.02563	0.02486	0.02265
35 thy DQ180333 Thy	0.00263	0.02201	0.01673	0.03485	0.03001	0.02922	0.02359
36 thy EF495063 Thy	0.02659	0.02457	0.02780	0.03840	0.03266	0.03185	0.02953
37 thy EF527269 Hni	0.02781	0.02572	0.02893	0.03608	0.03216	0.03136	0.03223
38 thy EF612464 Thy	0.00196	0.01659	0.01223	0.02887	0.02422	0.02346	0.01809

Maximum-likelihood distance matrix (continued)

	32	33	34	35	36	37	38
32 thy AF540903 Thy	-						
33 thy AY472084 Thy	0.00738	-					
34 thy AY472085 Thy	0.02761	0.02841	-				
35 thy DQ180333 Thy	0.00460	0.00668	0.02525	-			
36 thy EF495063 Thy	0.03061	0.03318	0.02384	0.02987	-		
37 thy EF527269 Hni	0.03272	0.03355	0.02983	0.03119	0.03605	-	
38 thy EF612464 Thy	0.00131	0.00599	0.01966	0.00262	0.02476	0.02684	-
